# Impacts of *ABCG2* loss of function variant (p. Gln141Lys, c.421 C > A, rs2231142) on lipid levels and statin efficiency: a systematic review and meta-analysis

**DOI:** 10.1186/s12872-024-03821-2

**Published:** 2024-04-08

**Authors:** Yang Liu, Yuan Chen, Baozhu Wei, Hang Li, Yuanyuan Peng, Zhi Luo

**Affiliations:** 1 Department of Cardiology, Zhongnan Hospital of Wuhan University, Wuhan University, Wuhan, China; 2 Department of Endocrinology, China Resources and WISCO General Hospital, Wuhan, China; 3 Department of Cardiology, Union Hospital, Tongji Medical College, Huazhong University of Science and Technology, Wuhan, China; 4 Institute of Myocardial Injury and Repair, Wuhan University, Wuhan, China; 5 Department of Geratology, Zhongnan Hospital of Wuhan University, Wuhan University, Wuhan, China; 6 Department of Cardiology, Suining Central Hospital, Suining, Sichuan 629000 China

**Keywords:** ABCG2, rs2231142, Dyslipidemia, Statin, Coronary artery disease

## Abstract

**Background:**

The latest evidence indicates that ATP-binding cassette superfamily G member 2 (ABCG2) is critical in regulating lipid metabolism and mediating statin or cholesterol efflux. This study investigates whether the function variant loss within *ABCG2* (rs2231142) impacts lipid levels and statin efficiency.

**Methods:**

PubMed, Cochrane Library, Central, CINAHL, and ClinicalTrials.gov were searched until November 18, 2023.

**Results:**

Fifteen studies (34,150 individuals) were included in the analysis. The A allele [Glu141Lys amino acid substitution was formed by a transversion from cytosine (C) to adenine (A)] of rs2231142 was linked to lower levels of high-density lipoprotein cholesterol (HDL-C), and higher levels of low-density lipoprotein cholesterol (LDL-C) and total cholesterol (TC). In addition, the A allele of rs2231142 substantially increased the lipid-lowering efficiency of rosuvastatin in Asian individuals with dyslipidemia. Subgroup analysis indicated that the impacts of rs2231142 on lipid levels and statin response were primarily in Asian individuals.

**Conclusions:**

The *ABCG2* rs2231142 loss of function variant significantly impacts lipid levels and statin efficiency. Preventive use of rosuvastatin may prevent the onset of coronary artery disease (CAD) in Asian individuals with dyslipidemia.

**Supplementary Information:**

The online version contains supplementary material available at 10.1186/s12872-024-03821-2.

## Background

The ABCG2 protein, also called breast cancer resistance protein (BCRP), mediates cellular efflux of a variety of xenobiotics, including statins, anticancer agents, cytotoxic agents, and antibiotics [1, 2], thus contributing to multidrug resistance [3].

The *ABCG2* gene is located on the long arm of human chromosome 4 (4q22-q23), including 16 exons. rs2231142 is located in the fifth exon, formed by a transversion from cytosine (C) to adenine (A) with the amino acid replacement of glutamine (Gln) by lysine (Lys) in the BCRP polypeptide. The C and A alleles encode high and low activity of ABCG2, respectively. The ATPase activity in Sf9 insect cells transfected with Glu141Lys ABCG2 was 1.8-fold lower than in cells transfected with wild-type ABCG2 [4]. In addition, heterozygous individuals (CA) with an *ABCG2* nonsense mutation on one allele had a 50% decrease in the protein expression of ABCG2 compared to homozygous wild-type individuals (CC) [5].

Emerging evidence indicates that ABCG2/BCRP is related to lipid metabolism. For instance, Taylor et al. [6] and Jackson et al. [7], using cryo-electron microscopy insight into the structure of the ABCG2 transporter [6, 7], found that cholesterol molecules were located in the multidrug-binding pocket of ABCG2. It indicated that ABCG2 might be involved in cholesterol transport or metabolism. Notably, this speculation was verified in Scharenberg et al. [8, 9] studies, whereby ABCG2 is critical in mediating cholesterol and Hoechst 33,342 (lipophilic dye) efflux. In addition, treatment of BeWo cells with cholesterol sequestrant methyl-β-cyclodextrin (MβCD) relocated ABCG2 protein into a higher density non-lipid raft fractions [10]. In contrast, repleting the cells with cholesterol restored ABCG2 localization to lipid raft-containing fractions [10].

ABCG2 plays a vital role in clearing cholesterol and maintaining lipid metabolism homeostasis [6–10]. Since the expression levels [11] and function [12, 13] of ABCG2 are primarily determined by the rs2231142 variant, it is tempting to speculate that the rs2231142 variant may result in dyslipidemia. This study was conducted to investigate this hypothesis.

A series of studies indicated that the rs2231142 variant might influence the pharmacokinetics of statin. For instance, a clinical trial [14] revealed that the rs2231142 variant significantly improved the pharmacokinetics of fluvastatin and simvastatin. In contrast, a systematic review with meta-analysis [15] showed that the rs2231142 variant significantly increased the pharmacokinetics of rosuvastatin. In addition, a systematic review without meta-analysis [16] reported that the impact of rs2231142 on statin pharmacokinetics was stronger in rosuvastatin than in fluvastatin and simvastatin. Taken together, these results [14–16] suggested that the rs2231142 variant might alter the lipid-lowering efficiency of statin. Here, we conducted this study to investigate this hypothesis.

## Methods

This meta-analysis follows the Preferred Reporting Items for Systematic Reviews and Meta-analyses (PRISMA) [17].

### Literature search

A comprehensive literature search was performed from January 10, 2021 to November 18, 2023 using PubMed, Cochrane Library, Central, CINAHL, and ClinicalTrials.gov. The following keywords were used in the search: [“ATP-binding cassette superfamily G member 2,” “ABCG2,” “BCRP,” “breast cancer resistance protein”] AND [“polymorphism,” “mutation,” “variation,” “mutant,” “variant,” “SNP”] OR [“single nucleotide polymorphism”] AND [“lipids,” “circulating lipids,” “blood lipids,” “plasma lipids,” “serum lipids,” “lipid profile”] OR [“triglycerides,” “total cholesterol,” “low-density lipoprotein cholesterol,” “high-density lipoprotein cholesterol”] AND/OR [“statin,” “statin response,” “statin therapy,” “lipid-lowering therapy,” “lipid-lowering efficiency,” “HMG-CoA reductase inhibitors,” “hydrophilic statins,” “hydrophobic statins,” “lipophilic statins,” “rosuvastatin,” “pravastatin,” “atorvastatin,” “simvastatin,” “pitavastatin,” “lovastatin,” “fluvastatin”]. Additionally, the reference lists of all eligible studies were manually retrieved to search for additional literature.

### Inclusion criteria

The inclusion criteria for the impacts of rs2231142 on lipid levels include: (1) The study investigated the relationship between rs2231142 and lipid levels. (2) The study provided at least three of four parameters in lipid profiles [ie, triglycerides (TG), TC, LDL-C, and HDL-C]. (3) The study provided the number of rs2231142 genotype. (4) The study provided mean lipid levels with standard deviation (SD) or standard errors (SE) by the genotype of rs2231142. The inclusion criteria for the impact of rs2231142 on statin efficiency include: (1) The study investigated the association between rs2231142 and lipid-lowering response. (2) The study provided the mean percentage change of lipid levels with SD or SE by the genotype of rs2231142.

### Data extraction

Three authors (YL, YC, and BW) independently extracted the data using standardized data extraction sheets. The discrepancy in data collected was resolved by consensus or a discussion with the senior author (ZL). Main data points included study details (first author’s name, year, country, ethnicity, gender, genotype number, genotyping methods, type of disease, study design, follow-up period, and total sample size), mean lipid levels with SD or SE by the genotype of rs2231142 and mean percentage change of lipid levels with SD or SE by the genotype of rs2231142.

### Data analysis

All extracted data were unified as mean ± SD. For instance, if the studies presented their data as mean ± SE, the SD = SE* $\sqrt{n}$ was used to get SD. The percentage change of lipid levels in different genotypes was calculated by the formula *p* = [(after treatment- baseline)/baseline]*100%. The standardized mean difference (SMD) and 95% confidence interval (CI) were used to evaluate the differences in lipid levels between the genotype of rs2231142. The mean difference (MD) and 95% CI were used to evaluate the mean percentage change of lipid levels between the genotype of rs2231142. Since most of the included studies presented lipid levels and the percentage change of lipid levels in a dominant model (CA + AA vs. CC), a dominant model was adopted to ensure adequate statistical power. All statistical tests were conducted with the Cochrane Collaboration meta-analysis software, Review Manager 5.4. *P* < 0.05 was considered as statistically significant.

### Subgroup analysis

Subgroup analyses were performed in healthy Asian individuals, Asian individuals with dyslipidemia and/or gout, and Caucasian individuals with dyslipidemia and/or gout, etc. In some studies, the individuals were divided into more than one subpopulation (e.g., the individuals originated from different ethnicities or sexes). Each subpopulation was regarded as an independent comparison in this study.

### Evaluation of heterogeneity

Heterogeneity was tested by the *I*^2^ statistic and Cochran’s χ2-based Q statistic. If heterogeneity was significant (*I*^2^ > 50%, *P* ≤ 0.05), a random-effects model (DerSimonian-Laird method) was used to calculate the results [18]. Otherwise, a fixed-effects model (Mantel-Haenszel method) was adopted (*I*^2^ < 50%, *P* > 0.05). In addition, the Galbraith plot was employed to detect the potential sources of heterogeneity. To eliminate the impact of heterogeneity on results, all results were recalculated after excluding studies with heterogeneity.

### Sensitivity analysis

Sensitivity analysis was conducted in this meta-analysis whereby these comparisons were excluded one by one, and the analysis was performed again after omitting each comparison. Suppose the results in any comparison changed substantially to alter the results from significant to non-significant or vice versa. The absence of such a phenomenon usually indicates the robustness of analysis results.

### Publication bias test

The publication bias among the included studies was evaluated by Begg’s funnel plot and Egger’s linear regression test [19]. The funnel plots were asymmetric when there were publication biases and symmetric in case of no publication bias.

## Results

### Study selection

**Fig. 1:**
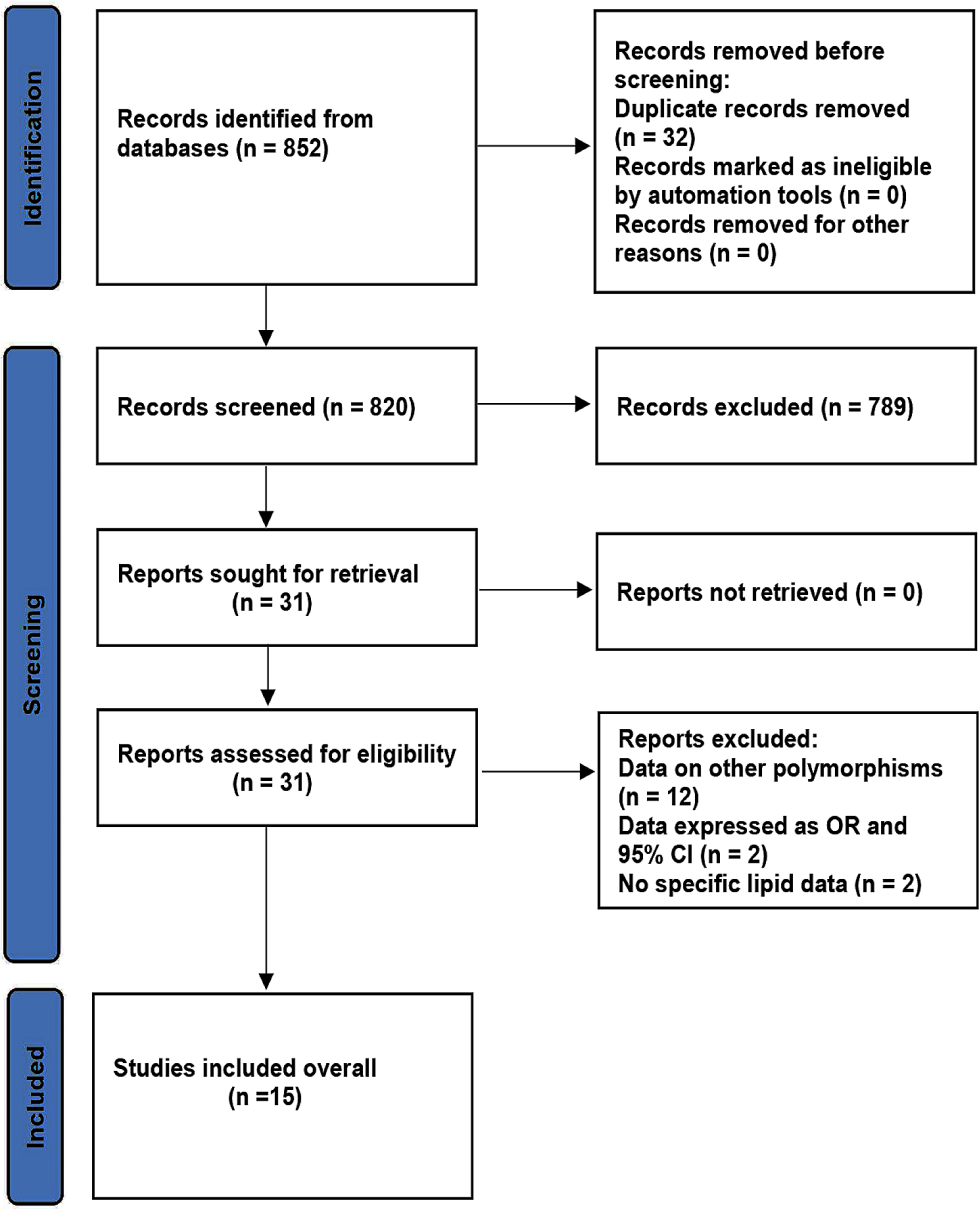
Flow diagram of the studies selection process

The details of the study selection are summarized in Fig. 1.

### Characteristics of the included studies

The present study included 15 studies in a total of 34,150 individuals. The characteristics of the included studies are presented in Additional file 1: Table S1. The blood lipid levels by the genotype of the *ABCG2* rs2231142 polymorphism are presented in Additional file 1: Table S2. The lipid-lowering response to statin by the genotype of the *ABCG2* rs2231142 polymorphism is presented in Additional file 1: Table S3. The sensitivity analysis of the *ABCG2* rs2231142 variant with blood lipid levels is presented in Additional file 1: Figure S1. The sensitivity analysis of the *ABCG2* rs2231142 variant with lipid-lowering response to statin is presented in Additional file 1: Figure S2. The Begg funnel plot evaluating publication bias for the impacts of the *ABCG2* rs2231142 variant on blood lipid levels is presented in Additional file 1: Figure S3. The Begg funnel plot evaluating publication bias for the impacts of the *ABCG2* rs2231142 variant on lipid-lowering response to statin is presented in Additional file 1: Figure S4.

### Impacts of rs2231142 on lipid levels

**Fig. 2:**
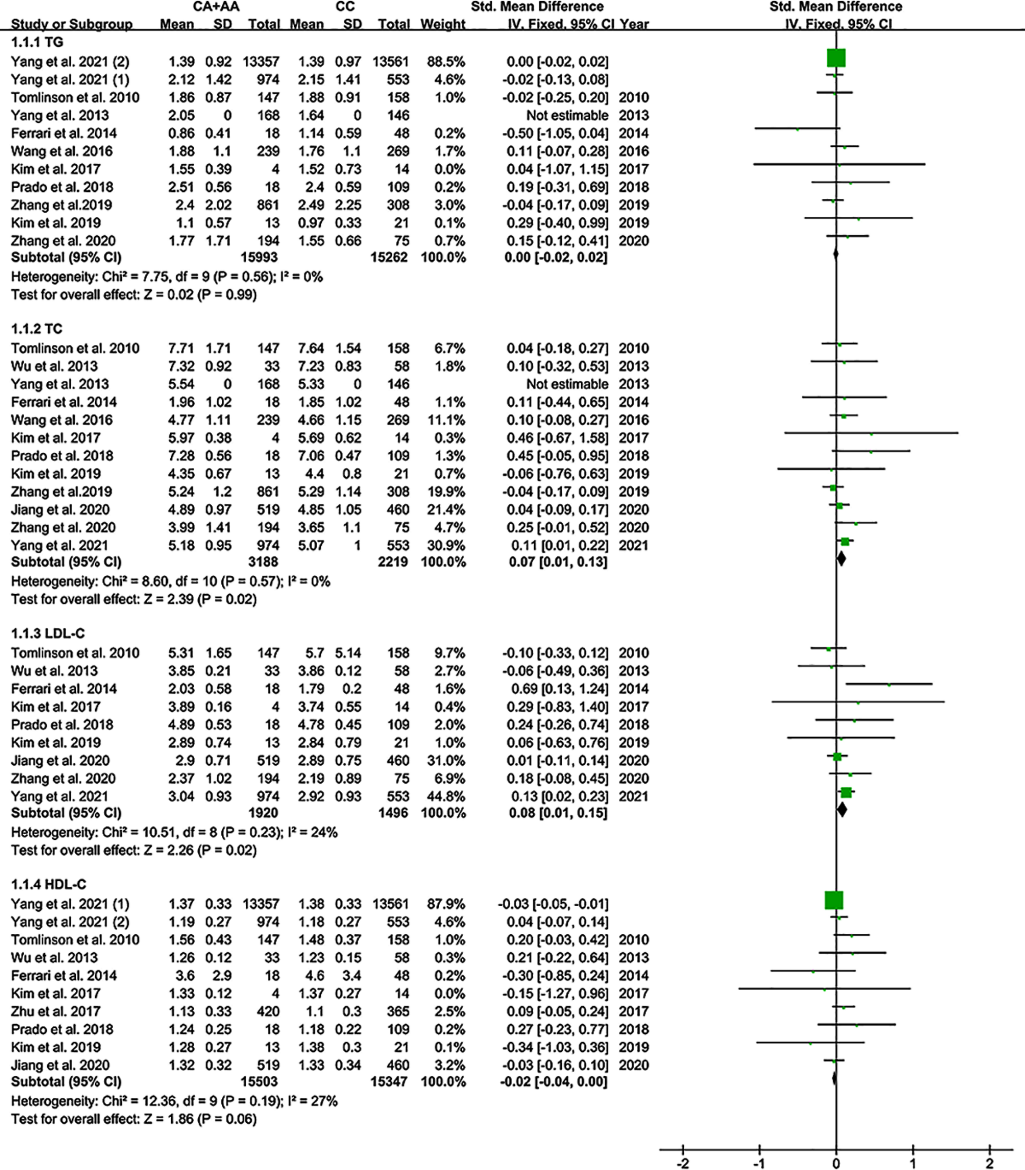
Forest plot of the meta-analysis between the *ABCG2* rs2231142 variant and lipid profiles (TG: mmol/L, TC: mmol/L, LDL-C: mmol/L, HDL-C: mmol/L)

All the results stated below were the data excluded heterogeneity. The consistent findings for rs2231142 on lipid levels were increased LDL-C and TC levels and decreased HDL-C levels (Fig. 2). Subgroup analysis indicated that the impact of rs2231142 on LDL-C levels was significant in healthy Asian individuals and in Caucasian individuals with dyslipidemia or gout (Additional file 2: Table 1). In addition, the impacts of rs2231142 on TC and HDL-C levels were significant in healthy Asian individuals (Additional file 2: Table 1). In contrast, a marginally significant impact of rs2231142 on HDL-C levels was observed in Asian individuals and in individuals with dyslipidemia (Additional file 2: Table 1).

### Impact of rs2231142 on statin efficiency

**Fig. 3:**
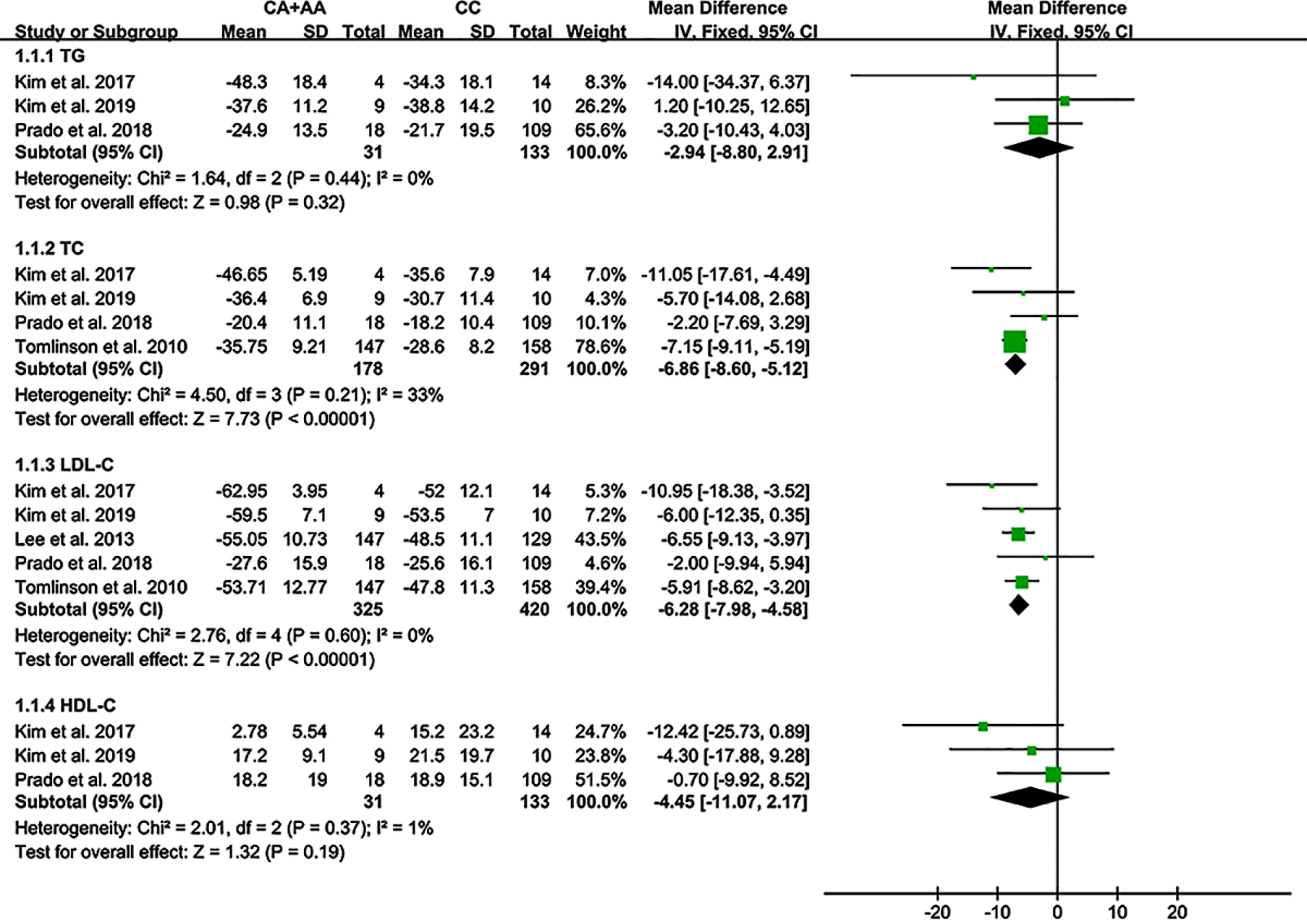
Forest plot of the meta-analysis between the *ABCG2* rs2231142 variant and statin response (TG: %, TC: %, LDL-C: %, HDL-C: %)

The A allele of rs2231142 substantially increased the lipid-lowering efficiency of statin (Fig. 3). Subgroup analysis indicated that the impact of rs2231142 on statin efficiency was significant in Asian individuals with dyslipidemia or rosuvastatin therapy (Additional file 3: Table 2).

### Evaluation of heterogeneity

In analyzing the impacts of rs2231142 on lipid levels (Table 1), significant heterogeneity was detected (Table 1). However, the recalculated results did not change substantially after excluding the studies with heterogeneity (see Table 1 for more details). It indicates the reliability of the analysis results.

### Sensitivity analysis

Sensitivity analysis indicated that one, three, and one comparisons might affect the impacts of rs2231142 on TC, LDL-C, and HDL-C levels (see Figure S1 for more details). One comparison might affect the impact of rs2231142 on statin efficiency (see Figure S2 for more details). However, the recalculated results remained relatively the same after omitting these comparisons (see Figure S1 and Figure S2 for more details). It indicates the robustness of the analysis results.

### Publication bias test

This meta-analysis confirmed no publication bias, which was demonstrated by the Egger linear regression test (see Figure S3 and Figure S4 for more details).

## Discussion

This study demonstrated that the A allele of rs2231142 was linked to lower levels of HDL-C and higher levels of LDL-C and TC (Fig. 2). In contrast, an ameliorated lipid-lowering response to rosuvastatin was observed in Asian individuals with dyslipidemia (Fig. 3).

Several potential mechanisms could be proposed to explain the impacts of the rs2231142 variant on lipid levels and statin efficiency. At first, ABCG2 is critical in removing excess cholesterol [6–9]; the loss of function variant in the *ABCG2* gene (rs2231142) leads to the inhibition of ABCG2 function [12, 13], thus resulting in dyslipidemia. Secondly, ATP binding cassette transporter 1 (ABCB1) is known to influence lipid levels; a recent study [20] indicates that ABCG2 may indirectly influence lipid levels by regulating ABCB1 expression. Since the expression levels of ABCG2 are primarily determined by rs2231142 [11], it indicates that the rs2231142 variant may induce dyslipidemia by modulating ABCB1 expression. Additionally, the decline of ABCG2 activity associated with the rs2231142 variant [11–13] increases the absorption of statin in the gastrointestinal tract while decreasing drug efflux in biliary ducts [21]. The dual effects of enhanced absorption and reduced hepatic clearance lead to drug accumulation in the systemic circulation and increase the lipid-lowering efficiency of statin [21].

Dyslipidemia is characterized by decreased HDL-C levels and increased TG, TC, and LDL-C [22]. Since dyslipidemia is one of the most critical risk factors for CAD and accounts for at least 50% of population-attributable risk [23], it is tempting to speculate that the increased CAD risk associated with the rs2231142 A allele [24–26] may stem from increased LDL-C and TC levels and decreased HDL-C levels (Fig. 2). The impacts of rs2231142 on LDL-C, TC, and HDL-C levels were significant in Asian individuals (Table 1), indicating that Asian individuals with the rs2231142 A allele had an increased risk of suffering CAD. In addition, a considerable impact [the SMD values (SMD = 0.44, 95% CI = 0.07 to 0.82, *P* = 0.02) that calculated in Caucasian individuals were much larger than those calculated in other subpopulations, please see Table 1-right panel-recalculated results that eliminated heterogeneity for more details] of rs2231142 on LDL-C levels was observed in Caucasian individuals with dyslipidemia and/or gout (Table 1), indicating that Caucasian individuals with dyslipidemia and/or gout were at high risk of CAD.

According to the 2018 ACC/AHA [27], the 2019 ESC/EAS [28], and the Adult Treatment Panel III (ATP III) cholesterol guidelines [29], LDL-C was considered the major cause of CAD and treated as the primary target for therapy, while other lipids were used as the secondary or supplementary therapeutic targets. In the present study, the rs2231142 A allele substantially increased lipid-lowering response (i.e., lowered LDL-C and TC levels) to rosuvastatin (Table 2) in Asian individuals with dyslipidemia, indicating that the preventive use of rosuvastatin may prevent the onset of CAD in Asian individuals with dyslipidemia. However, whether statin can reduce the risk of CAD in Caucasian and African individuals remains unknown due to the limited number of studies (Table 2). Further studies on Caucasian and African individuals are certainly needed.

No genome-wide association study (GWAS) has investigated the impact of rs2231142 on lipid levels. However, a GWAS study by Dehghan et al. [30] indicated that the rs2231142 A allele significantly elevated serum uric acid (SUA) levels. Since genetically determined SUA levels were linked to dyslipidemia [31], it was plausible to detect that rs2231142 was associated with dyslipidemia (Table 1). Recently, two GWAS studies [32, 33] revealed that the A allele of rs2199936 reduced LDL-C levels in individuals with rosuvastatin. Since rs2231142 (Chromosome: 4; Position: 89,052,323) [31] and rs2199936 (Chromosome: 4; Position: 89,264,355) [33] are located in the same genetic region of the *ABCG2* gene and are in linkage disequilibrium [32], it is tempting to hypothesize that rs2231142 and rs2199936 may have a similar biological function [32–34]. As expected, the A allele of rs2231142 reduced LDL-C levels in individuals with rosuvastatin (Table 2).

Considering these findings, future research should focus on expanding the understanding of the role of the *ABCG2* rs2231142 loss of function variant in pharmacogenetic testing. Specifically, investigating its impact on drug metabolism and response to different medications can provide valuable insights for personalized medicine and optimizing treatment strategies.

The present systematic review and meta-analysis have several strengths. (1) All results are recalculated after excluding studies with heterogeneity (Tables 1 and 2), which advances the preciseness of conclusions drawn in this paper. (2) rs2231142 has a significant impact on lipid levels (Table 1), which indicates that the association between rs2231142 and CAD [23–25] is mediated, at least partly, by the impacts of rs2231142 on lipid levels (Table 1). (3) The rs2231142 A allele substantially enhances lipid-lowering response (especially LDL-C) to rosuvastatin (Table 2) in Asian individuals with dyslipidemia, indicating that the preventive use of rosuvastatin may prevent the onset of CAD in Asian individuals with dyslipidemia. (4) Genetic screening of the rs2231142 variant is meaningful for the early prevention of dyslipidemia and CAD. One major limitation should be noted when interpreting the results of this study. The interactions of rs2231142 with other variant locus or environmental factors on lipid levels have yet to be investigated in the present study due to the lack of original data from the included studies. In other words, more precise results could have been gained if more detailed individual data were available, or the stratification analyses based on environmental factors, such as diet, exercise, smoking, etc., were performed.

## Conclusions

The *ABCG2* rs2231142 loss of function variant significantly impacts lipid levels and statin efficiency. Preventive use of rosuvastatin may prevent the onset of CAD in Asian individuals with dyslipidemia.

### Electronic supplementary material

Below is the link to the electronic supplementary material.

Supplementary Material 1

Supplementary Material 2

Supplementary Material 3

## Data Availability

All data generated or analysed during this study are included in this published article and its Additional file 1–3.

## Abbreviations

ABCG2: ATP-binding cassette superfamily G member 2
ABCB1: ATP binding cassette transporter 1
BCRP: breast cancer resistance protein
TG: triglycerides
TC: total cholesterol
LDL-C: low-density lipoprotein cholesterol
HDL-C: high-density lipoprotein cholesterol
CAD: coronary artery disease
MβCD: methyl-β-cyclodextrin
SUA: serum uric acid
Gln: glutamine
Lys: lysine
SD: standard deviation
SE: standard errors
SMD: standardized mean difference
CI: confidence interval
GWAS: genome-wide association study
PRISMA: Preferred Reporting Items for Systematic Reviews and Meta-analyses
