# Supplementary material for: Impacts of ABCG2 loss of function variant (p. Gln141Lys, c.421 C > A, rs2231142) on lipid levels and statin efficiency: a systematic review and meta-analysis
Source: BMC Cardiovasc Disord. 2024 Apr 8;24:202. doi: 10.1186/s12872-024-03821-2 (PMC11000409; doi:10.1186/s12872-024-03821-2)
Supplement: Supplementary file 1 — Supplementary Material 1 [file 12872_2024_3821_MOESM1_ESM.doc]

**Supplemental Tables:**

*Table S1* **Characteristics of the included studies.**

*Table S2* **Blood lipid levels by the genotype of the *ABCG2* rs2231142 polymorphism.**

*Table S3* **Lipid-lowering response to statin by the genotype of the *ABCG2* rs2231142 polymorphism.**

**Supplemental Figures:**

*Figure S1* **Sensitivity analysis of the *ABCG2* rs2231142 variant with blood lipid levels (A: triglyceride levels; B: total cholesterol levels; C: low-density lipoprotein cholesterol levels; D: high-density lipoprotein cholesterol levels).**

*Figure S2* **Sensitivity analysis of the *ABCG2* rs2231142 variant with lipid-lowering response to statin (A: triglyceride levels; B: total cholesterol levels; C: low-density lipoprotein cholesterol levels; D: high-density lipoprotein cholesterol levels).**

*Figure S3* **Begg funnel plot evaluating publication bias for the impacts of the *ABCG2* rs2231142 variant on blood lipid levels (A: triglyceride levels; B: total cholesterol levels; C: low-density lipoprotein cholesterol levels; D: high-density lipoprotein cholesterol levels).**

*Figure S4* **Begg funnel plot evaluating publication bias for the impacts of the *ABCG2* rs2231142 variant on lipid-lowering response to statin (A: triglyceride levels; B: total cholesterol levels; C: low-density lipoprotein cholesterol levels; D: high-density lipoprotein cholesterol levels).**

*Table S1* **Characteristics of the included studies.**

| **First author, reference** | **Year** | **Country** | **Ethnicity** | **Gender** | **Study population** | **Outcomes** | **Treatment**  **protocol** | **Duration of follow-up** |
| --- | --- | --- | --- | --- | --- | --- | --- | --- |
| Zhang D [S1] | 2020 | China | Asian | M/F | Patients with dyslipidemia and T2DM | TG/TC/LDL-C/HDL-C | - | - |
| Zhang K [S2] | 2019 | China | Asian | M | Patients with gout | TG/TC | - | - |
| Prado Y [S3] | 2018 | Chile | Caucasian | M/F | Patients with dyslipidemia | TG/TC/LDL-C/HDL-C | Atorvastatin:  10mg/day | 4 weeks |
| Zhu L [S4] | 2017 | China | Asian | M/F | Patients with CAD | LDL-C/HDL-C | - | - |
| Jiang Y [S5] | 2020 | China | Asian | M/F | Healthy individuals | TC/LDL-C/HDL-C | - | - |
| Kim TE [S6] | 2017 | Korea | Asian | M/F | Patients with dyslipidemia | TG/TC/LDL-C/HDL-C | Rosuvastatin:  20mg/day | 8 weeks |
| Tomlinson B [S7] | 2010 | China | Asian | M/F | Patients with dyslipidemia | TG/TC/LDL-C/HDL-C | Rosuvastatin:  10mg/day | > 4 weeks |
| Yang HJ [S8] | 2021 | Korea | Asian | M/F | Healthy individuals | TG/TC/LDL-C/HDL-C | - | - |
| Yang HJ [S8] | 2021 | Korea | Asian | M/F | Healthy individuals | TG/TC/LDL-C/HDL-C | - | - |
| Zheng C [S9] | 2016 | China | Asian | M/F | Patients with gout and control individuals | TG/TC/LDL-C/HDL-C | - | - |
| Wang JD [S10] | 2016 | China | Asian | M/F | Patients with gout | TG/TC | - | - |
| Yang HY [S11] | 2013 | China | Asian | M/F | Patients with gout | TG/TC | - | - |
| Wu JC [S12] | 2013 | China | Asian | M/F | Patients with dyslipidemia | TG/TC/LDL-C/HDL-C | - | - |
| Kim Y [S13] | 2019 | Korea | Asian | M/F | Patients with dyslipidemia | TG/TC/LDL-C/HDL-C | Rosuvastatin:  20mg/day | 3 weeks |
| Ferrari M [S14] | 2014 | Italy | Caucasian | M/F | Patients with statin-induced elevated serum creatine kinase | TG/TC/LDL-C/HDL-C | - | - |
| Lee HK [S15] | 2013 | China | Asian | M/F | Patients with dyslipidemia | TG/TC/LDL-C/HDL-C | Rosuvastatin:  10mg/day | > 4 weeks |

M: male; F: female; BC: breast carcinoma; CAD: coronary artery disease; T2DM: type 2 diabetes mellitus; TG: triglycerides; TC: total cholesterol; LDL-C: low-density lipoprotein cholesterol; HDL-C: high-density lipoprotein cholesterol.

***Table S2* Blood lipid levels by the genotype of the *ABCG2* rs2231142 polymorphism.**

| **First author,**  **reference** | **Number** | |  | **TG, mmol/L** | |  | **TC, mmol/L** | |  | **LDL-C, mmol/L** | |  | **HDL-C, mmol/L** | |
| --- | --- | --- | --- | --- | --- | --- | --- | --- | --- | --- | --- | --- | --- | --- |
|  | **CC** | **CA+AA** |  | **CC** | **CA+AA** |  | **CC** | **CA+AA** |  | **CC** | **CA+AA** |  | **CC** | **CA+AA** |
| Zhang D [S1] | 75 | 194 |  | 1.55±0.66 | 1.77±1.71 |  | 3.65±1.10 | 3.99±1.41 |  | 2.19±0.89 | 2.37±1.02 |  | 1.00±0.27 | 1.09±0.31 |
| Zhang K [S2] | 308 | 861 |  | 2.49±2.25 | 2.4±2.02 |  | 5.29±1.14 | 5.24±1.2 |  | - | - |  | - | - |
| Prado Y [S3] | 109 | 18 |  | 2.4±0.59 | 2.51±0.56 |  | 7.06±0.47 | 7.28±0.56 |  | 4.78±0.45 | 4.89±0.53 |  | 1.18±0.22 | 1.24±0.25 |
| Zhu L [S4] | 365 | 420 |  | - | - |  | - | - |  | 2.4±0.7 | 2.8±0.87 |  | 1.1±0.3 | 1.13±0.33 |
| Jiang Y [S5] | 460 | 519 |  | - | - |  | 4.85±1.05 | 4.89±0.97 |  | 2.89±0.75 | 2.9±0.71 |  | 1.33±0.34 | 1.32±0.32 |
| Kim TE [S6] | 14 | 4 |  | 1.52±0.73 | 1.55±0.39 |  | 5.69±0.62 | 5.97±0.38 |  | 3.74±0.55 | 3.89±0.16 |  | 1.37±0.27 | 1.33±0.12 |
| Tomlinson B [S7] | 158 | 147 |  | 1.88±0.91 | 1.86±0.87 |  | 7.64±1.54 | 7.71±1.71 |  | 5.70±5.14 | 5.31±1.65 |  | 1.48±0.37 | 1.56±0.43 |
| Yang HJ [S8] | 13561 | 13357 |  | 1.39±0.97 | 1.39±0.92 |  | 5.09±0.92 | 5.09±0.91 |  | 3.08±0.84 | 3.08±0.83 |  | 1.38±0.33 | 1.37±0.33 |
| Yang HJ [S8] | 553 | 974 |  | 2.15±1.41 | 2.12±1.42 |  | 5.07± 1 | 5.18±0.95 |  | 2.92±0.93 | 3.04±0.93 |  | 1.18±0.27 | 1.19±0.27 |
| Zheng C [S9] | 93 | 202 |  | 1.76±1.61 | 2.53±1.3 |  | 1.29±0.28 | 5.59±2.19 |  | 3.48±3.52 | 4.92±2.78 |  | 1.30±0.65 | 1.97±1.49 |
| Wang JD [S10] | 269 | 239 |  | 1.76±1.10 | 1.88±1.10 |  | 4.66±1.15 | 4.77±1.11 |  | - | - |  | - | - |
| Yang HY [S11] | 146 | 168 |  | 1.64±0 | 2.05±0 |  | 5.33±0 | 5.54±0 |  | - | - |  | - | - |
| Wu JC [S12] | 58 | 33 |  | 2.21±0.23 | 2.36±0.26 |  | 7.23±0.83 | 7.32±0.92 |  | 3.86±0.12 | 3.85±0.21 |  | 1.23±0.15 | 1.26±0.12 |
| Kim Y [S13] | 21 | 13 |  | 0.97±0.33 | 1.1±0.57 |  | 4.4±0.8 | 4.35±0.67 |  | 2.84±0.79 | 2.89±0.74 |  | 1.38±0.3 | 1.28±0.27 |
| Ferrari M [S14] | 48 | 18 |  | 1.14±0.59 | 0.86±0.41 |  | 1.85±1.02 | 1.96±1.02 |  | 1.79±0.2 | 2.03±0.58 |  | 4.6±3.4 | 3.6±2.9 |

*ABCG2:* ATP-binding cassette superfamily G member 2; TG: triglycerides; TC: total cholesterol; LDL-C: low-density lipoprotein cholesterol; HDL-C: high-density lipoprotein cholesterol.

***Table S3* Lipid-lowering response to statin by the genotype of the *ABCG2* rs2231142 polymorphism.**

| **First author,**  **reference** | **Number** | |  | **TG (% change)** | |  | **TC (% change)** | |  | **LDL-C (% change)** | |  | **HDL-C (% change)** | |
| --- | --- | --- | --- | --- | --- | --- | --- | --- | --- | --- | --- | --- | --- | --- |
|  | **CC** | **CA+AA** |  | **CC** | **CA+AA** |  | **CC** | **CA+AA** |  | **CC** | **CA+AA** |  | **CC** | **CA+AA** |
| Prado Y [S3] | 109 | 18 |  | -21.7±19.5 | -24.9±13.5 |  | -18.2±10.4 | -20.4±11.1 |  | -25.6±16.1 | -27.6±15.9 |  | 18.9±15.1 | 18.2±19 |
| Kim TE [S6] | 14 | 4 |  | -34.3±18.1 | -48.28±18.41 |  | -35.6±7.9 | -46.65±5.19 |  | -52±12.1 | -62.95±3.95 |  | 15.2±23.2 | 2.78±5.54 |
| Tomlinson B [S7] | 158 | 147 |  | - | - |  | -28.6±8.2 | -35.75±9.21 |  | -47.8±11.3 | -53.71±12.77 |  | - | - |
| Kim Y [S13] | 10 | 9 |  | -38.8±14.2 | -37.6±11.2 |  | -30.7±11.4 | -36.4±6.9 |  | -53.5±7 | -59.5±7.1 |  | 21.5±19.7 | 17.2±9.1 |
| Lee HK [S15] | 129 | 147 |  | - | - |  | - | - |  | -48.5±11.1 | -55.05±10.73 |  | - | - |

*ABCG2:* ATP-binding cassette superfamily G member 2; TG: triglycerides; TC: total cholesterol; LDL-C: low-density lipoprotein cholesterol; HDL-C: high-density lipoprotein cholesterol.

**
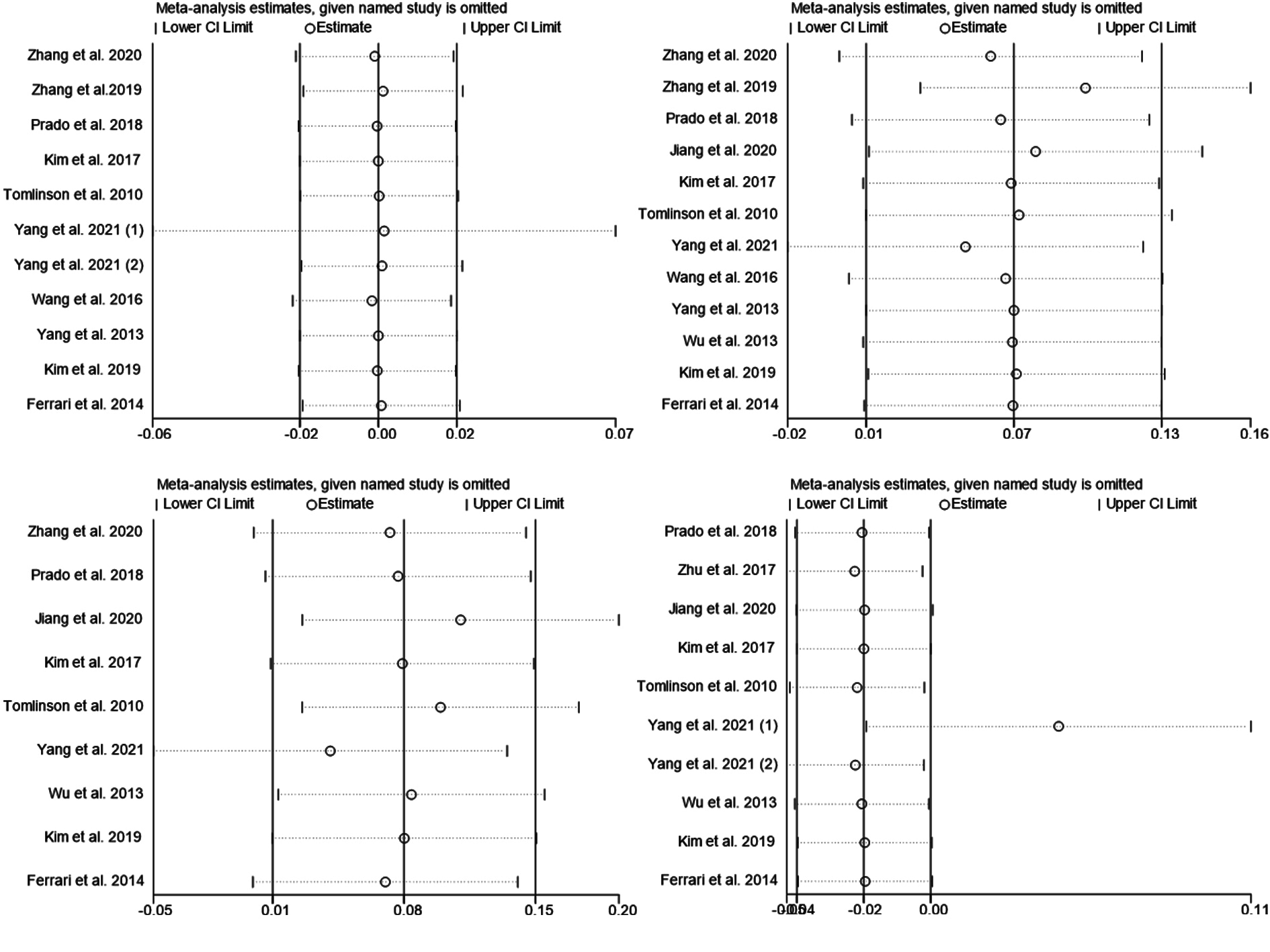
**

*Figure S1* **Sensitivity analysis of the *ABCG2* rs2231142 variant with blood lipid levels (A: triglyceride levels; B: total cholesterol levels; C: low-density lipoprotein cholesterol levels; D: high-density lipoprotein cholesterol levels).**

*
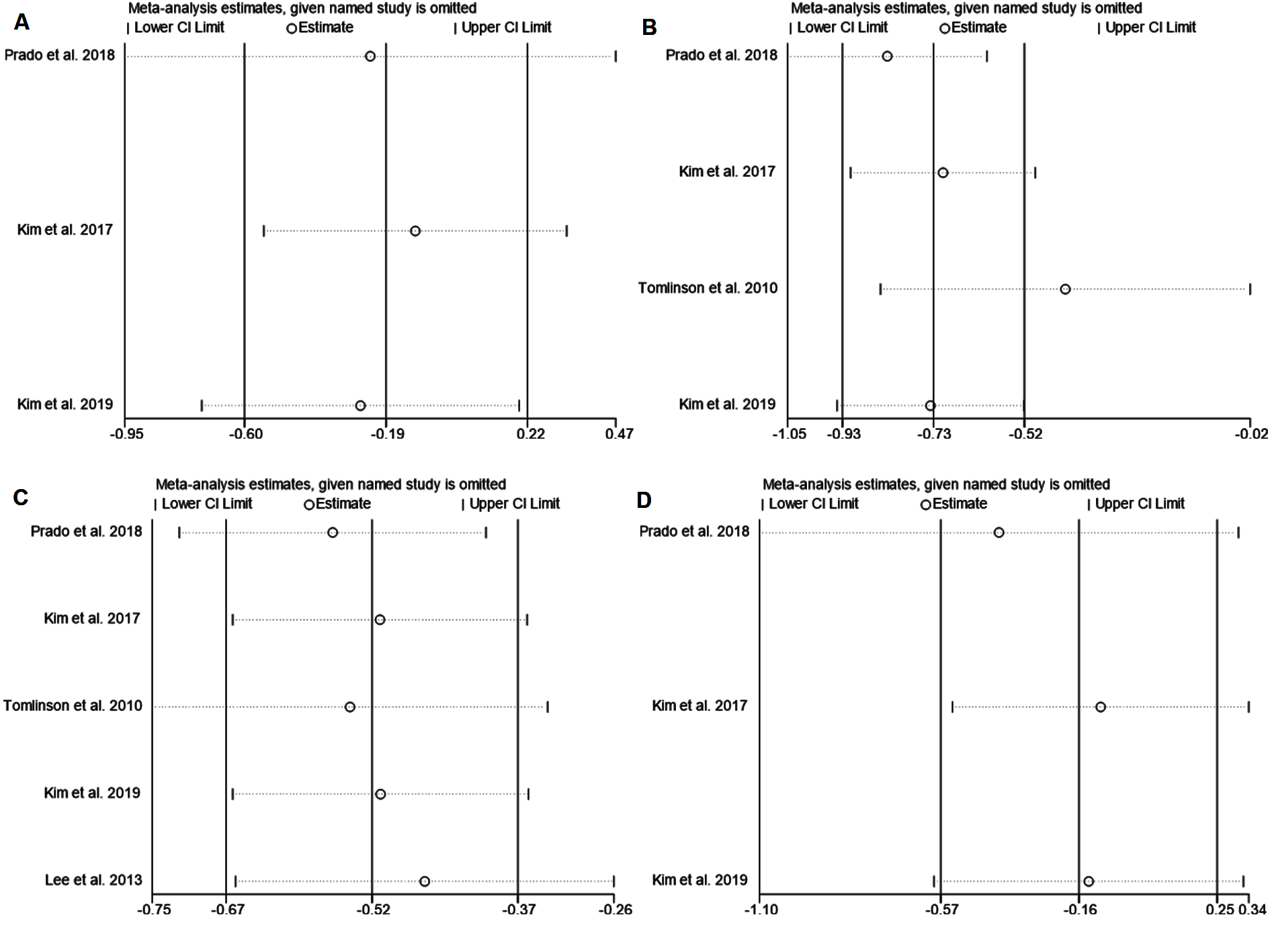
*

*Figure S2* **Sensitivity analysis of the *ABCG2* rs2231142 variant with lipid-lowering response to statin (A: triglyceride levels; B: total cholesterol levels; C: low-density lipoprotein cholesterol levels; D: high-density lipoprotein cholesterol levels).**

**
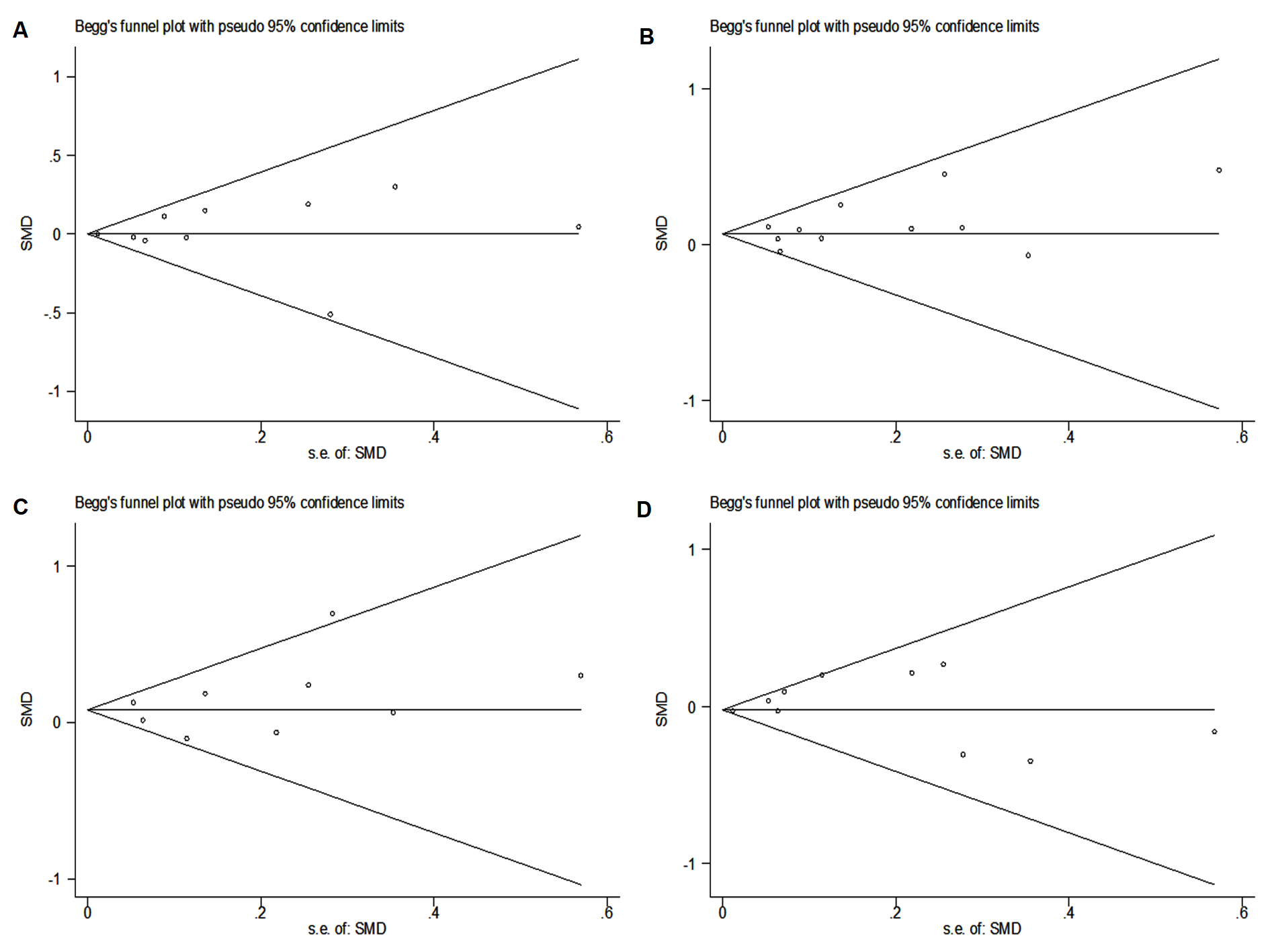
**

*Figure S3* **Begg funnel plot evaluating publication bias for the impacts of the *ABCG2* rs2231142 variant on blood lipid levels (A: triglyceride levels; B: total cholesterol levels; C: low-density lipoprotein cholesterol levels; D: high-density lipoprotein cholesterol levels).**

**
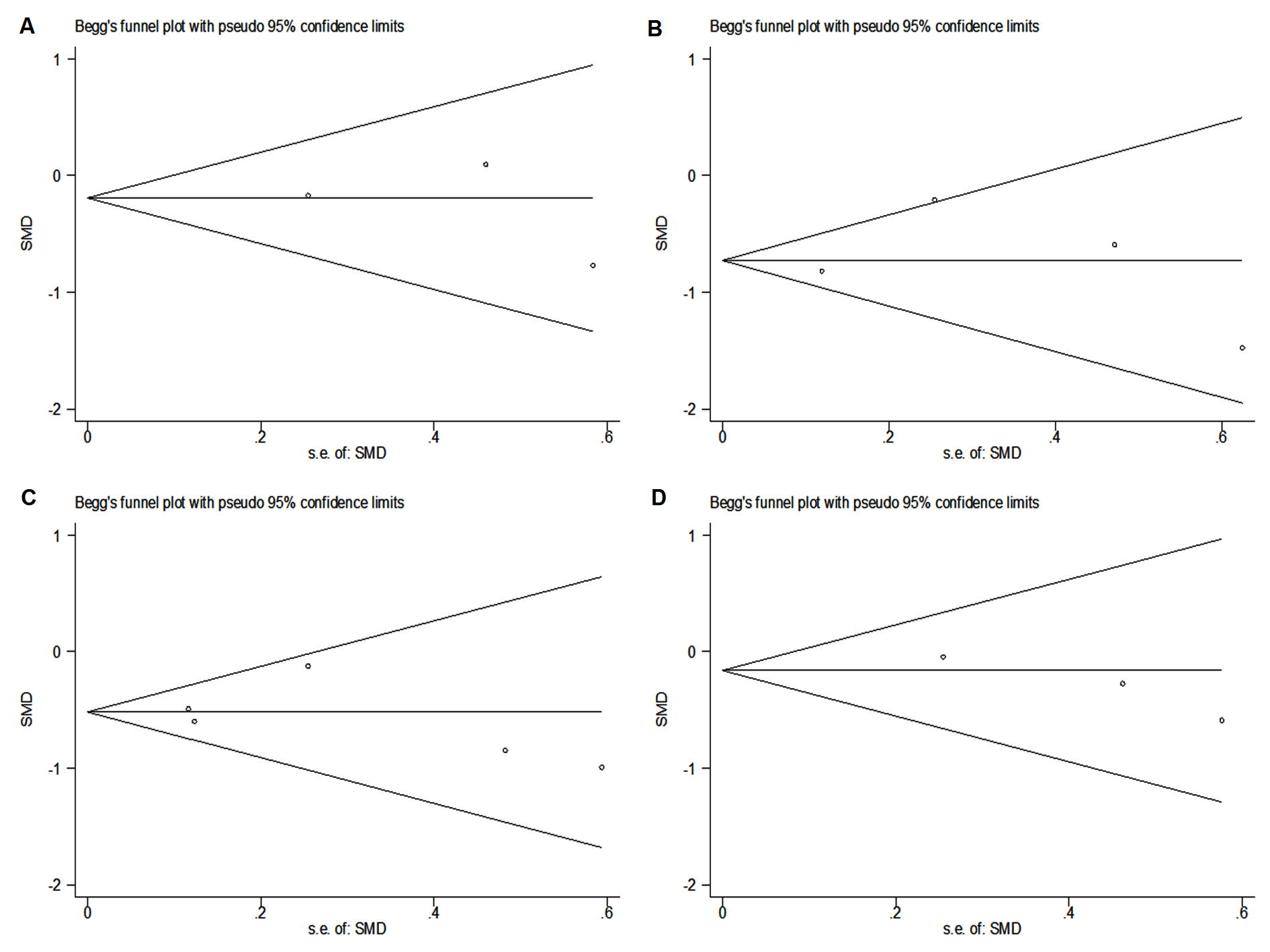
**

*Figure S4* **Begg funnel plot evaluating publication bias for the impacts of the *ABCG2* rs2231142 variant on lipid-lowering response to statin (A: triglyceride levels; B: total cholesterol levels; C: low-density lipoprotein cholesterol levels; D: high-density lipoprotein cholesterol levels).**

**References**

S1. Zhang D, Ding Y, Wang X, Xin W, Du W, Chen W, Zhang X, Li P. Effects of ABCG2 and SLCO1B1 gene variants on inflammation markers in patients with hypercholesterolemia and diabetes mellitus treated with rosuvastatin. Eur J Clin Pharmacol. 2020;76(7):939-946. doi: 10.1007/s00228-020-02882-4.

S2. Zhang K, Li C. ABCG2 gene polymorphism rs2231142 is associated with gout comorbidities but not allopurinol response in primary gout patients of a Chinese Han male population. Hereditas. 2019;156:26. doi: 10.1186/s41065-019-0103-y.

S3. Prado Y, Zambrano T, Salazar LA. Transporter genes ABCG2 rs2231142 and ABCB1 rs1128503 polymorphisms and atorvastatin response in Chilean subjects. J Clin Pharm Ther. 2018;43(1):87-91. doi: 10.1111/jcpt.12607.

S4. Zhu L, Ji X, Jiang L, Zhu Y, Xu Y, Jiang Q, Bao J, Ye J, Sheng H, Yu H. Utility of genetic variants to predict prognosis in coronary artery disease patients receiving statin treatment. Int J Clin Exp Pathol. 2017;10(8):8795-8803.

S5. Jiang Y, Ge JY, Zhang YY, Wang FF, Ji Y, Li HY. The relationship between elevated serum uric acid and arterial stiffness in a healthy population. Vascular. 2020;28(4):494-501. doi: 10.1177/1708538120913721.

S6. Kim TE, Shin D, Gu N, Jung BH, Kim J, Cho YM, Yu KS, Cho JY. The Effect of Genetic Polymorphisms in SLCO2B1 on the Lipid-Lowering Efficacy of Rosuvastatin in Healthy Adults with Elevated Low-Density Lipoprotein. Basic Clin Pharmacol Toxicol. 2017;121(3):195-201. doi: 10.1111/bcpt.12826.

S7. Tomlinson B, Hu M, Lee VW, Lui SS, Chu TT, Poon EW, Ko GT, Baum L, Tam LS, Li EK. ABCG2 polymorphism is associated with the low-density lipoprotein cholesterol response to rosuvastatin. Clin Pharmacol Ther. 2010;87(5):558-62. doi: 10.1038/clpt.2009.232.

S8. Yang HJ, Liu M, Kim MJ, Park S. The haplotype of SLC2A9_rs3733591, PKD2_rs2725220 and ABCG2_rs2231142 increases the hyperuricaemia risk and alcohol, chicken and processed meat intakes and smoking interact with its risk. Int J Food Sci Nutr. 2021;72(3):391-401. doi: 10.1080/09637486.2020.1807474.

S9. Zheng C, Yang H, Wang Q, Rao H, Diao Y. Association analysis of five SNP variants with gout in the Minnan population in China. Turk J Med Sci. 2016;46(2):361-7. doi: 10.3906/sag-1409-58.

S10. Wang JD, Yu LL, Huang DY, Shi SX, Zhao MM, Fang LL, Li F. The relationship between polymorphism of rs2231142 of ABCG2 gene and primary gout in Zhe Nan population. Basic ＆ Clinical Medicine. 2016;36(4):503-507. doi: 1001-6325(2016)04-0503-05.

S11. Yang HY, Xu CC, Wang QY, You YQ, Rao HC, Zheng CN, Diao Y. Correlational study between ABCG2 gene single nucleotide polymorphism and primary gout in the Min Nan population. Rheumatism and Arthritis. 2013;2(1): 24-28.

S12. Wu JC. Effect of ABCG2 genetic polymorphism on the lipid-lowering role of atorvastatin. Chin J Clin Pharm Therap. 2014; 19 (8): 882-884.

S13. Kim Y, Yoon S, Choi Y, Yoon SH, Cho JY, Jang IJ, Yu KS, Chung JY. Influence of OATP1B1 and BCRP polymorphisms on the pharmacokinetics and pharmacodynamics of rosuvastatin in elderly and young Korean subjects. Sci Rep. 2019;9(1):19410. doi: 10.1038/s41598-019-55562-4.

S14. Ferrari M, Guasti L, Maresca A, Mirabile M, Contini S, Grandi AM, Marino F, Cosentino M. Association between statin-induced creatine kinase elevation and genetic polymorphisms in SLCO1B1, ABCB1 and ABCG2. Eur J Clin Pharmacol. 2014;70(5):539-47. doi: 10.1007/s00228-014-1661-6.

S15. Lee HK, Hu M, Lui SSh, Ho CS, Wong CK, Tomlinson B. Effects of polymorphisms in ABCG2, SLCO1B1, SLC10A1 and CYP2C9/19 on plasma concentrations of rosuvastatin and lipid response in Chinese patients. Pharmacogenomics. 2013;14(11):1283-94. doi: 10.2217/pgs.13.115.
