# Supplementary material for: Impacts of ABCG2 loss of function variant (p. Gln141Lys, c.421 C > A, rs2231142) on lipid levels and statin efficiency: a systematic review and meta-analysis
Source: BMC Cardiovasc Disord. 2024 Apr 8;24:202. doi: 10.1186/s12872-024-03821-2 (PMC11000409; doi:10.1186/s12872-024-03821-2)
Supplement: Supplementary file 2 — Supplementary Material 2 [file 12872_2024_3821_MOESM2_ESM.docx]

*Table 1* **Meta-analysis of the association between the *ABCG2* rs2231142 variant and blood lipid levels.**

| **Groups or subgroups** | **Subjects** | ***P*_H_** | **SMD (95% CI)** | ***P*_SMD_** |  | **Groups or subgroups** | **Subjects** | ***P*_H_** | **SMD (95% CI)** | ***P*_SMD_** |
| --- | --- | --- | --- | --- | --- | --- | --- | --- | --- | --- |
| ***Overall results*** | | | | |  | ***Recalculated results that eliminated heterogeneity*** | | | | |
| **TG** |  |  |  |  |  | **TG** |  |  |  |  |
| All individuals | 31,641 | <0.001 | 0.08 (-0.02-0.18)  04 | 0.10 |  | All individuals | 31,255 | 0.56 | 0.00 (-0.02-0.02)  04 | 0.99 |
| Asian individuals | 31,448 | <0.001 | 0.09 (-0.01-0.19) | 0.06 |  | Asian individuals | 31,062 | 0.78 | 0.00 (-0.02-0.02) | 0.95 |
| Healthy Asian individuals | 28,445 | 0.70 | -0.00 (-0.02-0.02) | 0.93 |  | Healthy Asian individuals | 28,445 | 0.70 | -0.00 (-0.02-0.02) | 0.93 |
| Asian individuals with dyslipidemia  and/or gout | 3,003 | <0.01 | 0.19 (0.01-0.37) | 0.04 |  | Asian individuals with dyslipidemia  and/or gout | 2,617 | 0.62 | 0.03 (-0.06-0.11) | 0.57 |
| Caucasian individuals with dyslipidemia  and/or gout | 193 | 0.07 | -0.15 (-0.84-0.53) | 0.66 |  | Caucasian individuals with dyslipidemia  and/or gout | 193 | 0.07 | -0.13 (-0.50-0.24) | 0.50 |
| Asian and Caucasian individuals with dyslipidemia | 910 | 0.14 | 0.21 (-0.07-0.50) | 0.41 |  | Asian and Caucasian individuals with dyslipidemia | 819 | 0.76 | 0.04 (-0.16-0.23) | 0.72 |
| Asian and Caucasian individuals with gout | 2,286 | 0.17 | 0.02 (-0.13-0.17) | 0.78 |  | Asian and Caucasian individuals with gout | 1,991 | 0.17 | 0.01 (-0.09-0.12) | 0.83 |
| **TC** |  |  |  |  |  | **TC** |  |  |  |  |
| All individuals | 32,620 | <0.001 | 0.29 (0.09-0.49)  04 | 0.01 |  | All individuals | 5,407 | 0.57 | 0.07 (0.01-0.13)  04 | 0.02 |
| Asian individuals | 32,427 | <0.001 | 0.29 (0.07-0.50) | 0.01 |  | Asian individuals | 5,214 | 0.60 | 0.07 (0.01-0.12) | 0.03 |
| Healthy Asian individuals | 29,424 | 0.03 | 0.04 (-0.03-0.12) | 0.25 |  | Healthy Asian individuals | 2,506 | 0.28 | 0.09 (0.02-0.15) | 0.01 |
| Asian individuals with dyslipidemia  and/or gout | 3,003 | <0.001 | 0.50 (-0.12-1.12) | 0.11 |  | Asian individuals with dyslipidemia  and/or gout | 2,708 | 0.09 | 0.04 (-0.03-0.10) | 0.27 |
| Caucasian individuals with dyslipidemia  and/or gout | 193 | 0.36 | 0.30 (-0.07-0.66) | 0.12 |  | Caucasian individuals with dyslipidemia  and/or gout | 193 | 0.36 | 0.30 (-0.07-0.66) | 0.12 |
| Asian and Caucasian individuals with dyslipidemia | 910 | 0.59 | 0.11 (-0.07-0.28) | 0.23 |  | Asian and Caucasian individuals with dyslipidemia | 910 | 0.59 | 0.11 (-0.07-0.28) | 0.23 |
| Asian and Caucasian individuals with gout | 1,991 | 0.21 | 0.02 (-0.12-0.15) | 0.83 |  | Asian and Caucasian individuals with gout | 1,991 | 0.21 | 0.01 (-0.09-0.11) | 0.89 |
| **LDL-C** |  |  |  |  |  | **LDL-C** |  |  |  |  |
| All individuals | 31,414 | <0.001 | 0.17 (0.04-0.31)  04 | 0.01 |  | All individuals | 3,416 | 0.23 | 0.08 (0.01-0.15)  04 | 0.02 |
| Asian individuals | 31,221 | <0.001 | 0.15 (0.01-0.29) | 0.04 |  | Asian individuals | 3,223 | 0.49 | 0.07 (0.00-0.14) | 0.05 |
| Healthy Asian individuals | 29,424 | 0.01 | 0.04 (-0.04-0.12) | 0.31 |  | Healthy Asian individuals | 2,506 | 0.16 | 0.13 (0.03-0.23) | 0.04 |
| Asian individuals with dyslipidemia  and/or gout | 1,012 | <0.01 | 0.14 (-0.09-0.36) | 0.22 |  | Asian individuals with dyslipidemia  and/or gout | 717 | 0.57 | 0.01 (-0.14-0.16) | 0.86 |
| Caucasian individuals with dyslipidemia  and/or gout | 193 | 0.23 | 0.45 (0.00-0.90) | 0.05 |  | Caucasian individuals with dyslipidemia  and/or gout | 193 | 0.23 | 0.44 (0.07-0.82) | 0.02 |
| Asian and Caucasian individuals with dyslipidemia | 910 | 0.75 | -0.03 (-0.21-0.15) | 0.73 |  | Asian and Caucasian individuals with dyslipidemia | 910 | 0.75 | -0.03 (-0.21-0.15) | 0.73 |
| **HDL-C** |  |  |  |  |  | **HDL-C** |  |  |  |  |
| All individuals | 31,414 | <0.001 | 0.10 (0.00-0.19)  04 | 0.05 |  | All individuals | 30,850 | 0.19 | -0.02 (-0.04-0.00)  04 | 0.06 |
| Asian individuals | 31,221 | <0.001 | 0.10 (0.00-0.20) | 0.05  18 |  | Asian individuals | 30,657 | 0.18 | -0.02 (-0.04-0.00) | 0.06 |
| Healthy Asian individuals | 29,424 | 0.47 | -0.03 (-0.05--0.00) | 0.02 |  | Healthy Asian individuals | 29,424 | 0.46 | -0.03 (-0.05--0.00) | 0.02 |
| Asian individuals with dyslipidemia  and/or gout | 1,012 | 0.16 | 0.26 (0.08-0.45) | <0.01 |  | Asian individuals with dyslipidemia  and/or gout | 448 | 0.47 | 0.15 (-0.04-0.34) | 0.11 |
| Caucasian individuals with dyslipidemia  and/or gout | 193 | 0.13 | -0.01 (-0.57-0.55) | 0.98 |  | Caucasian individuals with dyslipidemia  and/or gout | 193 | 0.13 | -0.01 (-0.57-0.55) | 0.98 |
| Asian and Caucasian individuals with dyslipidemia | 910 | 0.61 | 0.17 (-0.01-0.34) | 0.06 |  | Asian and Caucasian individuals with dyslipidemia | 641 | 0.61 | 0.17 (-0.01-0.34) | 0.06 |

ABCG2: ATP-binding cassette superfamily G member 2; SMD: standardized mean difference; CI: confidence interval; TG: triglycerides (mmol/L); TC: total cholesterol (mmol/L); LDL-C: low-density lipoprotein cholesterol (mmol/L); HDL-C: high-density lipoprotein cholesterol (mmol/L). *P*_H_: *P* for heterogeneity.
