# Supplementary material for: Impacts of ABCG2 loss of function variant (p. Gln141Lys, c.421 C > A, rs2231142) on lipid levels and statin efficiency: a systematic review and meta-analysis
Source: BMC Cardiovasc Disord. 2024 Apr 8;24:202. doi: 10.1186/s12872-024-03821-2 (PMC11000409; doi:10.1186/s12872-024-03821-2)
Supplement: Supplementary file 3 — Supplementary Material 3 [file 12872_2024_3821_MOESM3_ESM.docx]

*Table 2* **Meta-analysis of the association between the ABCG2 rs2231142 variant and statin response.**

| **Groups or subgroups** | **Subjects** | ***P*_H_** | **MD (95% CI)** | ***P*_MD_** |
| --- | --- | --- | --- | --- |
| **TG** |  |  |  |  |
| Asian and Caucasian individuals with dyslipidemia | 164 | 0.44 | -2.94 (-8.80-2.91)  04 | 0.32 |
| Asian individuals with dyslipidemia | 37 | 0.20 | -2.45 (-12.42-7.53) | 0.63 |
| Asian individuals with Rosuvastatin therapy | 37 | 0.20 | -2.45 (-12.42-7.53) | 0.63 |
| **TC** |  |  |  |  |
| Asian and Caucasian individuals with dyslipidemia | 469 | 0.21 | -6.86 (-8.60--5.12)  04 | <0.001 |
| Asian individuals with dyslipidemia | 342 | 0.49 | -7.39 (-9.22--5.55)  04 | <0.001 |
| Asian individuals with Rosuvastatin therapy | 342 | 0.49 | -7.39 (-9.22--5.55)  04 | <0.001 |
| **LDL-C** |  |  |  |  |
| Asian and Caucasian individuals with dyslipidemia | 1,070 | 0.60 | -6.28 (-7.98--4.58)  04 | <0.001 |
| Asian individuals with dyslipidemia | 925 | 0.66 | -6.49 (-8.23--4.74)  04 | <0.001 |
| Asian individuals with Rosuvastatin therapy | 925 | 0.66 | -6.49 (-8.23--4.74)  04 | <0.001 |
| **HDL-C** |  |  |  |  |
| Asian and Caucasian individuals with dyslipidemia | 164 | 0.37 | -4.45 (-11.07-2.17)  04 | 0.19 |
| Asian individuals with dyslipidemia | 37 | 0.40 | -8.44 (-17.95-1.06) | 0.08  18 |
| Asian individuals with Rosuvastatin therapy | 37 | 0.40 | -8.44 (-17.95-1.06) | 0.08  18 |

ABCG2: ATP-binding cassette superfamily G member 2; MD: mean difference; CI: confidence interval; TG: triglycerides (%); TC: total cholesterol (%); LDL-C: low-density lipoprotein cholesterol (%); HDL-C: high-density lipoprotein cholesterol (%). *P*_H_: *P* for heterogeneity.
